# Case Report: Sinonasal plasmablastic lymphoma in a human immunodeficiency virus-negative elderly woman diagnosed by immunohistochemistry

**DOI:** 10.3389/fmed.2026.1925274

**Published:** 2026-08-26

**Authors:** Savitri M. Nerune, Sayandeep K. Das, Shivshankar Ajur, Ishan Garg

**Affiliations:** 1 Department of Pathology, Shri B. M. Patil Medical College Hospital and Research Centre, BLDE (Deemed to be University), Vijayapura, Karnataka, India; 2 Department of Otorhinolaryngology, Shri B. M. Patil Medical College Hospital and Research Centre, BLDE (Deemed to be University), Vijayapura, Karnataka, India

**Keywords:** Epstein–Barr virus, high-grade sinonasal malignancy, human immunodeficiency virus-negative, immunohistochemistry, kappa light-chain restriction, maxillary sinus, plasmablastic lymphoma, sinonasal lymphoma

## Abstract

**Background:**

Plasmablastic lymphoma is a rare, highly aggressive mature B-cell lymphoma with terminal B-cell/plasma-cell differentiation. It usually arises in the oral cavity in patients with human immunodeficiency virus infection or other forms of immunodeficiency, whereas primary sinonasal involvement in an apparently immunocompetent patient is uncommon. On hematoxylin-and-eosin examination, it may histomorphologically resemble poorly differentiated epithelial, melanocytic, and other hematolymphoid malignancies, necessitating a broad immunohistochemical panel for definitive diagnosis.

**Case presentation:**

A 75-year-old woman presented with a 1-month history of progressive left-sided nasal obstruction and watery nasal discharge, one episode of epistaxis 12 days before presentation, and reduced olfaction. She had no history of human immunodeficiency virus infection, organ transplantation, or systemic immunosuppression. Anterior rhinoscopy showed a mass occupying the left nasal cavity. Contrast-enhanced computed tomography demonstrated a heterogeneously enhancing, locally destructive soft-tissue lesion occupying the left maxillary sinus with extension into the left ethmoid and frontal sinuses and left nasal cavity, with erosion of the lamina papyracea and maxillary sinus walls. Biopsy showed a high-grade malignant round-cell/plasmablastic neoplasm with extensive necrosis. The main morphologic differentials included sinonasal undifferentiated carcinoma, non-keratinizing squamous cell carcinoma, amelanotic melanoma, non-Hodgkin lymphoma, and a plasma-cell neoplasm. Immunohistochemistry showed strong, diffuse expression of CD38, CD138, and MUM1; strong but focal nuclear PAX5 expression; focal leukocyte common antigen and Epstein–Barr virus positivity; kappa light-chain restriction; and a Ki-67 proliferation index of 98%−99%. Tumor cells were negative for CD20, pancytokeratin, AE1/AE3, S100, human herpesvirus 8, and lambda light chain. A final diagnosis of plasmablastic lymphoma was rendered. The patient received CHOP chemotherapy (cyclophosphamide, doxorubicin, vincristine, and prednisone) and had completed three cycles at 21-day intervals by the 5-month follow-up, with reduced nasal obstruction and overall symptomatic improvement. Post-treatment imaging was unavailable; therefore, radiological and metabolic response could not be assessed.

**Conclusion:**

This case highlights the need to include plasmablastic lymphoma in the differential diagnosis of an aggressive unilateral sinonasal mass, even in a human immunodeficiency virus-negative elderly patient, and emphasizes the decisive role of a broad immunohistochemical panel with light-chain assessment in resolving morphologic overlap with other high-grade sinonasal malignancies.

## Introduction

Plasmablastic lymphoma (PBL) is a rare and aggressive mature large B-cell lymphoma with plasmablastic morphology and terminal B-cell/plasma-cell differentiation recognized in the current World Health Organization classification of hematolymphoid tumors (1). The entity was first described by Delecluse et al. in 1997 (2) in human immunodeficiency virus (HIV)-associated oral cavity lymphomas with distinctive immunophenotypic features. Although PBL is classically linked to HIV infection, Epstein–Barr virus (EBV) infection, post-transplantation status, and other immunodeficiency settings, it can also occur in HIV-negative and apparently immunocompetent patients (3–5).

The oral cavity remains the prototypical site, but extraoral disease is well-documented. In a meta-analysis of reported cases, extranodal presentation was frequent, and HIV-negative PBL included a substantial subset of immunocompetent patients (5). Sinonasal PBL is particularly rare; a recent systematic review emphasized the limited number of reported sinonasal cases, the aggressive clinical behavior, and the lack of a universally accepted treatment approach for this anatomic presentation (6).

In routine surgical pathology practice, sinonasal PBL creates a diagnostic challenge because small biopsies may show necrosis, discohesive high-grade cells, and plasmablastic cytology that overlap with sinonasal undifferentiated carcinoma, non-keratinizing squamous cell carcinoma, amelanotic melanoma, other aggressive lymphomas, and plasmablastic plasma-cell myeloma. The present case is reported because an elderly HIV-negative woman presented with a unilateral locally destructive sinonasal mass, and the diagnosis was established only after integration of clinical information, radiology, histomorphology, immunohistochemistry, EBV testing, and light-chain restriction.

## Case description

A 75-year-old woman presented to the otorhinolaryngology service with progressive left-sided nasal obstruction and watery nasal discharge of 1-month duration. The symptoms were insidious in onset and gradually progressive. She also reported a single episode of epistaxis 12 days before presentation and a reduced sense of smell (Table 1). She had no history of diabetes mellitus, hypertension, smoking, or alcohol use. Family history was non-contributory. There was no documented history of HIV infection, organ transplantation, hematologic malignancy, long-term corticosteroid therapy, chemotherapy, or other systemic immunosuppression.

**Table 1:** Timeline of clinical course, diagnostic work-up, treatment, and follow-up.

Time point	Clinical event	Key findings/result	Diagnostic implication
1 month before presentation	Onset of left-sided nasal obstruction and watery nasal discharge	Insidious and progressive unilateral symptoms	Prompted otorhinolaryngology evaluation
12 days before presentation	Single episode of epistaxis with reduced olfaction	Bleeding and hyposmia in the setting of unilateral obstruction	Raised concern for an aggressive or neoplastic sinonasal lesion
At presentation	Clinical examination	Anterior rhinoscopy showed a mass occupying the left nasal cavity	Imaging and biopsy were indicated
Initial work-up	Baseline hematology, renal function, and viral serology	Hemoglobin 11.1 g/dL; other complete blood count parameters within normal limits; renal function normal; HIV, hepatitis B virus, and hepatitis C virus serology negative	No documented systemic immunodeficiency; baseline assessment before further management
Contrast-enhanced computed tomography	Radiologic assessment of paranasal sinuses	Heterogeneously enhancing left maxillary sinus mass extending to left ethmoid and frontal sinuses and left nasal cavity, with maxillary sinus wall destruction and lamina papyracea erosion	Locally aggressive lesion; malignant sinonasal neoplasm favored over benign polyp alone
Endoscopic biopsy	Tissue sampling of a polypoidal hypertrophic left nasal cavity lesion	High-grade malignant round-cell/plasmablastic neoplasm with extensive necrosis and inflammatory background	Broad differential diagnosis including undifferentiated carcinoma, non-keratinizing squamous cell carcinoma, amelanotic melanoma, non-Hodgkin lymphoma, and a plasma-cell neoplasm
Histopathology and immunohistochemistry	Integrated diagnostic assessment	Plasmablastic morphology; strong, diffuse CD38/CD138/MUM1 expression; strong but focal nuclear PAX5; focal CD45 and EBV positivity; kappa restriction; complete CD20 negativity; Ki-67 index 98%−99%	Final diagnosis of sinonasal plasmablastic lymphoma; atypical PAX5 expression interpreted within the complete clinicopathological panel
Post-diagnosis staging	Systemic staging and prognostic assessment	FDG PET-CT was not performed because the patient declined the investigation; Ann Arbor stage, serum LDH, and ECOG performance status were unavailable	Complete International Prognostic Index could not be calculated
5-month follow-up	Treatment and early outcome	CHOP chemotherapy; three cycles completed at 21-day intervals; reduction in nasal obstruction and overall symptomatic improvement; no post-treatment CT or FDG PET-CT available	Early clinical improvement; radiological and metabolic response could not be assessed

Anterior rhinoscopy revealed a mass occupying the left nasal cavity. Initial laboratory evaluation showed mild anemia with hemoglobin of 11.1 g/dL; the remaining complete blood count parameters were within normal limits. Renal function tests were normal. Serological tests for HIV, hepatitis B virus, and hepatitis C virus were negative.

**Figure 1:**
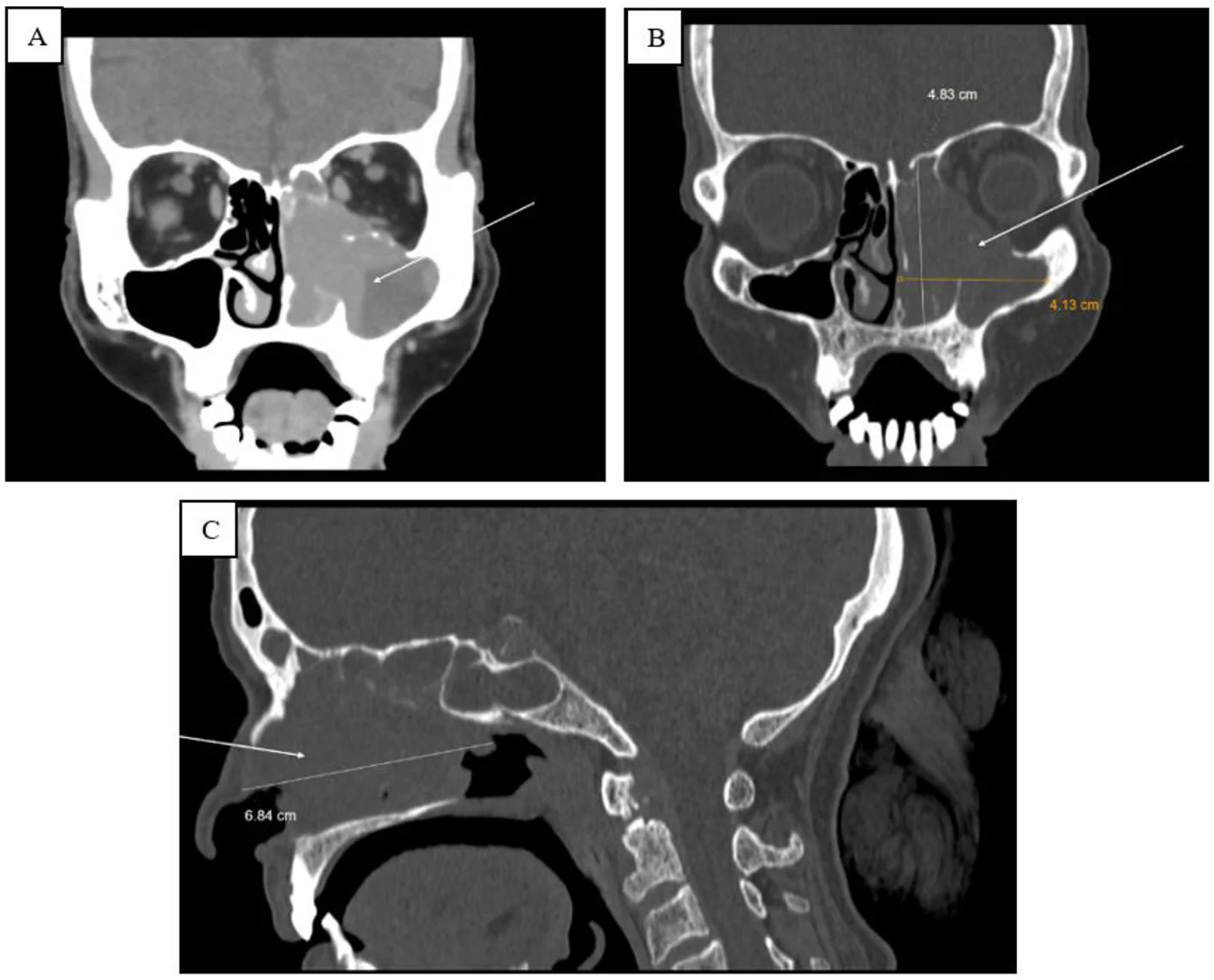
Contrast-enhanced computed tomography of the paranasal sinuses. **(A)** Coronal soft-tissue window. **(B)** Coronal bone window. The coronal images show a heterogeneously enhancing soft-tissue lesion occupying the left maxillary sinus, with extension into the left nasal cavity and adjacent sinuses. **(C)** Sagittal bone window showing the craniocaudal extent and posterior sinonasal involvement. Bone thinning/destruction and erosion of the lamina papyracea were noted radiologically.

Contrast-enhanced computed tomography (CECT) of the paranasal sinuses showed a heterogeneously enhancing soft-tissue lesion occupying the entire left maxillary sinus, measuring 6.84 × 4.83 × 4.13 cm, and extending into the left ethmoid sinus, left frontal sinus, and left nasal cavity. The lesion showed locally aggressive features, including thinning and destruction of the anterior and posterolateral walls of the left maxillary sinus and erosion of the left lamina papyracea. The CECT differential diagnosis included a chronic antrochoanal polyp and a malignant sinonasal neoplasm; however, the bony destruction and unilateral presentation favored a neoplastic process requiring tissue diagnosis (Figure 1).

Endoscopic evaluation demonstrated a polypoidal hypertrophic lesion arising from the left nasal cavity. A biopsy was obtained and submitted for histopathological examination.

## Diagnostic assessment

**Figure 2:**
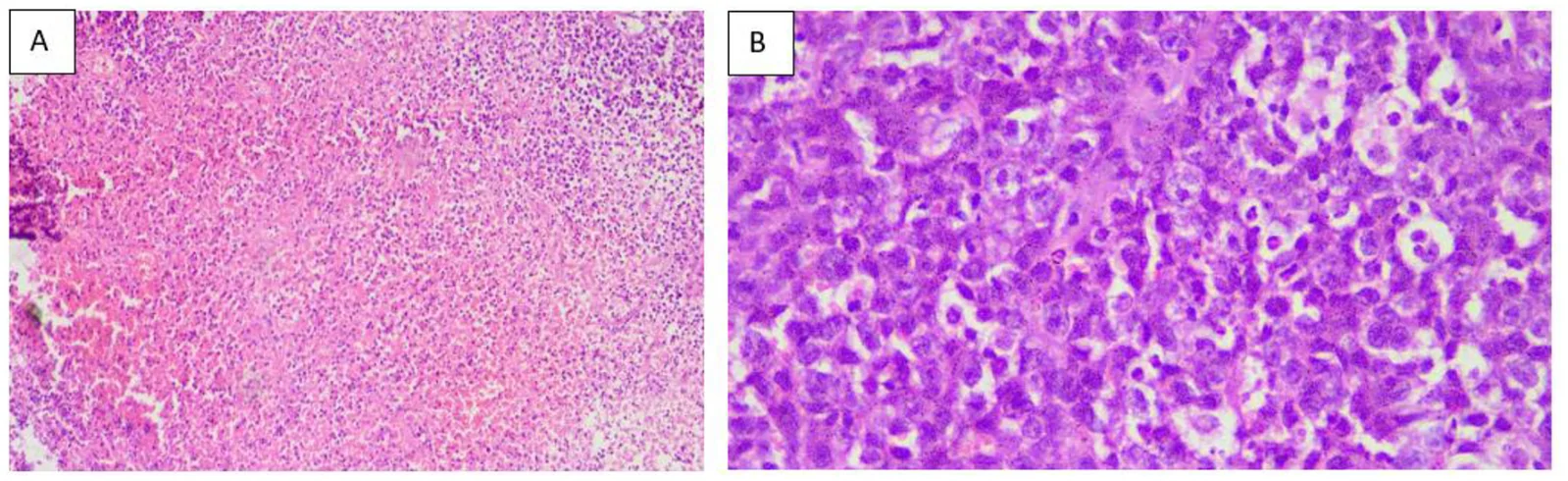
Hematoxylin-and-eosin-stained biopsy sections. **(A)** A × 100 view shows extensive areas of necrosis. **(B)** A × 400 view demonstrates large round-to-oval plasmablastic tumor cells with vesicular-to-hyperchromatic nuclei, prominent eosinophilic nucleoli, and high-grade cytologic features in an inflammatory background.

The biopsy showed tumor tissue arranged in nests, cords, and sheets. The tumor cells were large and round to oval, with hyperchromatic-to-vesicular nuclei, prominent eosinophilic nucleoli, and scant-to-moderate cytoplasm. Occasional bizarre cells with pleomorphic hyperchromatic nuclei and 4–6 mitotic figures per high-power field were also present (Figure 2). On morphology alone, the lesion was interpreted as a high-grade malignancy. The principal differential diagnoses were sinonasal undifferentiated carcinoma, non-keratinizing squamous cell carcinoma, amelanotic melanoma, non-Hodgkin lymphoma, and a plasma-cell neoplasm. Amelanotic melanoma was considered because of the prominent eosinophilic nucleoli, absence of visible melanin pigment, and sinonasal location.

**Figure 3:**
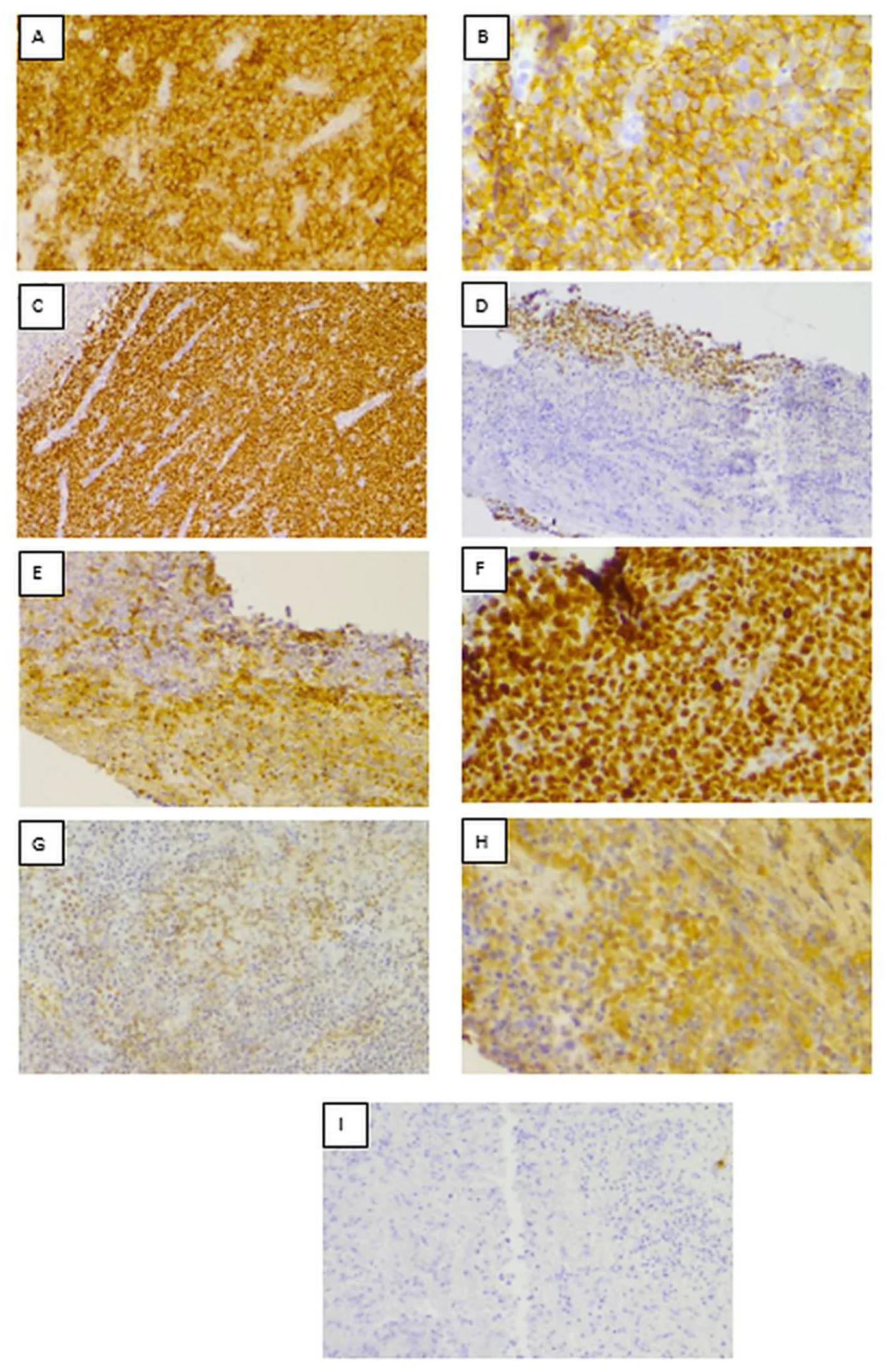
Immunohistochemical profile of the sinonasal mass. Tumor cells show **(A)** strong, diffuse membranous/cytoplasmic CD38 expression; **(B)** strong, diffuse membranous CD138 expression; **(C)** strong, diffuse nuclear MUM1 expression; **(D)** strong but focal nuclear PAX5 staining in a subset of tumor cells; **(E)** focal membranous leukocyte common antigen (CD45) staining; **(F)** nuclear Ki-67 labeling in approximately 98%−99% of tumor cells; **(G)** EBV positivity; **(H)** cytoplasmic kappa light-chain restriction; and **(I)** complete absence of CD20 staining in tumor cells.

A broad immunohistochemical panel was therefore applied. Tumor cells showed strong, diffuse expression of CD38, CD138, and MUM1; strong but focal nuclear PAX5 staining in a subset of morphologically identifiable tumor cells; and focal leukocyte common antigen (CD45) and EBV positivity. The Ki-67 proliferation index was extremely high at 98%−99%. Kappa light-chain restriction was present, whereas lambda was negative, supporting monoclonal plasma-cell differentiation. Tumor cells were completely negative for CD20 and were also negative for pancytokeratin, AE1/AE3, S100, and human herpesvirus 8. These findings excluded the major epithelial, melanocytic, and human herpesvirus 8-associated plasmablastic mimics and supported a final diagnosis of sinonasal plasmablastic lymphoma (Figure 3). Strong focal PAX5 expression is atypical for PBL and prompted careful consideration of EBV-positive diffuse large B-cell lymphoma with plasmablastic features. CD20 negativity alone does not exclude that differential; however, the integrated findings of plasmablastic morphology, diffuse plasma-cell-marker expression, light-chain restriction, EBV positivity, and a very high proliferation index favored PBL.

The biopsy phenotype, EBV positivity, high proliferation index, and clinical presentation favored PBL. Anaplastic Lymphoma Kinase (ALK) immunohistochemistry could not be performed because the residual biopsy tissue was limited. Fluorescence In Situ Hybridization (FISH) for MYC rearrangement was not performed, and CD79a and CD30 results were not available. Although EBV positivity made ALK-positive diffuse large B-cell lymphoma less typical, the absence of ALK testing means that this differential could not be formally excluded by immunohistochemistry. Because plasmablastic plasma-cell myeloma can show a closely overlapping immunophenotype, systemic correlation with staging imaging, bone marrow assessment, serum and urine protein electrophoresis, serum free light-chain assay, calcium level, lactate dehydrogenase, and myeloma-defining features is essential when clinically available.

Whole-body Fluorodeoxyglucose Positron Emission Tomography—Computed Tomography (FDG PET-CT) was not performed because the patient declined the investigation. Ann Arbor stage, serum lactate dehydrogenase level, and Eastern Cooperative Oncology Group performance status were unavailable; consequently, a complete International Prognostic Index score could not be calculated.

## Therapeutic intervention, follow-up, and outcomes

Following the diagnosis, the patient was referred to a medical oncology center. Chemotherapy was initiated after a 3-month interval using the CHOP regimen (cyclophosphamide, doxorubicin, vincristine, and prednisone). At the latest follow-up, 5 months after diagnosis, the patient had completed three cycles administered at 21-day intervals.

The patient reported clinical improvement, particularly a reduction in nasal obstruction and improvement in overall symptoms. No post-treatment CT or FDG PET-CT findings were available to the authors; therefore, radiological and metabolic response could not be assessed. The available follow-up remains short.

## Discussion

This case illustrates a rare presentation of PBL as a unilateral destructive sinonasal mass in an elderly HIV-negative woman. The clinical setting is notable because the classic prototype of PBL is an oral cavity tumor in a male HIV-positive patient, whereas HIV-negative patients are often older and may be either immunocompetent or affected by other immune dysregulation (2–5). Although no overt immunosuppressive condition was documented in this patient, advanced age may have contributed to relative immune dysregulation. The sinonasal tract adds another layer of diagnostic difficulty because many unilateral masses are inflammatory polyps, yet malignancy must not be overlooked. In a retrospective study of single-sided sinonasal masses, most lesions were benign, but 4.1% were malignant, supporting careful endoscopic examination and histopathologic assessment of unilateral lesions, especially when radiology shows bone destruction (7).

The sinonasal location in this case is uncommon but clinically important. Chen et al. recently reviewed sinonasal PBL and emphasized that reported cases span immunocompromised and immunocompetent adults, commonly require chemotherapy-based treatment, and remain difficult to manage because of rarity and absence of consensus therapy (6). Individual reports have described maxillary sinus PBL in an HIV-negative patient and nasal PBL in an HIV-negative immunocompetent patient, reinforcing that the diagnosis should not be dismissed when HIV serology is negative (8, 9). The present case expands this clinicopathological spectrum by documenting a destructive tumor involving the maxillary sinus with extension into the ethmoid and frontal sinuses and nasal cavity, with lamina papyracea erosion, in a 75-year-old woman.

The most important diagnostic lesson is that morphology alone is insufficient in a small sinonasal biopsy. Large cells with plasmablastic or immunoblastic features, necrosis, and an inflammatory background can mimic sinonasal undifferentiated carcinoma, poorly differentiated squamous cell carcinoma, melanoma, lymphoma, and plasma-cell neoplasms. In this case, negativity for pancytokeratin and AE1/AE3 argued against carcinoma, negativity for S100 argued against melanoma, and negativity for human herpesvirus 8 helped exclude human herpesvirus 8-associated large B-cell lymphoproliferations. Expression of CD38, CD138, and MUM1 confirmed plasmacytic differentiation; kappa light-chain restriction confirmed clonality; and the very high Ki-67 index matched the biologically aggressive nature of PBL.

The immunophenotype of PBL is distinctive but not always intuitive. PBL typically shows plasma-cell-marker expression with loss or weak expression of conventional B-cell markers, and it may show absent or weak leukocyte common antigen. This profile partly explains why PBL may be missed if the initial panel is restricted to epithelial and melanocytic markers. Studies comparing PBL with plasmablastic plasma-cell myeloma have shown that their immunophenotypes can be nearly identical, so clinical and laboratory correlation is mandatory (10). Immunohistochemical studies also show that combinations of absent or weak CD20/PAX5 with plasma-cell differentiation markers such as MUM1/IRF4, PRDM1/BLIMP1, XBP1, CD38, and CD138 are useful in recognizing the plasmablastic phenotype (11). In the present tumor, PAX5 staining was strong but focal and nuclear, an atypical finding that required careful consideration of EBV-positive diffuse large B-cell lymphoma with plasmablastic features. The diagnosis of PBL was favored only after integrating the complete CD20 negativity with the morphology, diffuse plasma-cell-marker expression, kappa light-chain restriction, EBV positivity, and the very high Ki-67 index. The unavailable ancillary studies remain an important diagnostic limitation.

EBV and MYC biology are central to the modern understanding of PBL. EBV is detected in a substantial proportion of PBL cases and may contribute to tumor survival and immune escape, although EBV negativity does not exclude the diagnosis (5, 12, 13). MYC rearrangements are among the most frequent cytogenetic abnormalities in PBL and are associated with the highly proliferative phenotype (12). In the present case, FISH for MYC rearrangement was not performed. ALK immunohistochemistry could not be completed because of limited residual tissue, and CD79a and CD30 results were unavailable. The Ki-67 index of 98%−99% nonetheless underscores the biological aggressiveness and the need for prompt systemic staging and oncologic management.

Therapeutic evidence for PBL remains limited because most data come from retrospective series, registry analyses, and case reports. Outcomes are generally poor compared with conventional diffuse large B-cell lymphoma, and several studies emphasize the influence of age, stage, EBV status, performance status, and treatment intensity on survival (3, 4, 14, 15). Cyclophosphamide, doxorubicin, vincristine, and prednisone alone has historically been considered inadequate for many patients, and more intensive approaches such as dose-adjusted Etoposide + Prednisone + Vincristine (Oncovin) + Cyclophosphamide + Doxorubicin (Hydroxydaunorubicin) (EPOCH), hyper-Cyclophosphamide, Vincristine, Adriamycin (Doxorubicin), Dexamethasone (CVAD)-based regimens, radiotherapy for localized disease, stem-cell transplantation in selected patients, and proteasome inhibitor-containing regimens have been explored. Bortezomib combined with EPOCH has shown encouraging activity in retrospective frontline experience, but there is no universally accepted standard regimen, especially for rare localized sinonasal presentations (16). In this case, CHOP was administered at the referral center. At 5 months after diagnosis, only early symptomatic improvement was documented, and no imaging-based response assessment was available.

The strength of this case is the integrated diagnostic work-up: a destructive unilateral sinonasal mass was assessed radiologically and histologically, followed by a broad immunohistochemical panel and light-chain evaluation that clarified the diagnosis. The main limitations are incomplete systemic staging; absence of FDG PET-CT; unavailable Ann Arbor stage, serum lactate dehydrogenase, and Eastern Cooperative Oncology Group (ECOG) performance status, which precluded calculation of the International Prognostic Index; incomplete myeloma-directed laboratory assessment; absence of ALK, CD79a, CD30, and MYC rearrangement testing; lack of post-treatment imaging; and the short 5-month follow-up. Despite these limitations, the case provides a practical take-away lesson for pathologists and clinicians: PBL should be considered in the differential diagnosis of high-grade unilateral sinonasal tumors in elderly HIV-negative patients, and early biopsy with a broad immunohistochemical panel is essential to prevent misclassification as carcinoma or melanoma.

## Conclusion

Sinonasal PBL is a rare diagnostic pitfall that can present as a locally destructive unilateral nasal cavity and paranasal sinus mass in an HIV-negative elderly patient. A broad immunohistochemical approach that includes epithelial, melanocytic, lymphoid, plasma-cell, viral, proliferation, and light-chain markers is critical for accurate diagnosis. Recognition of this entity has immediate clinical relevance because PBL requires systemic staging and lymphoma-directed management rather than treatment pathways for undifferentiated carcinoma, squamous cell carcinoma, melanoma, or benign sinonasal polyps.

## Data Availability

The original contributions presented in the study are included in the article/supplementary material, further inquiries can be directed to the corresponding authors.
